# Naringin‐inlaid silk fibroin/hydroxyapatite scaffold enhances human umbilical cord‐derived mesenchymal stem cell‐based bone regeneration

**DOI:** 10.1111/cpr.13043

**Published:** 2021-05-19

**Authors:** Zhi‐Hu Zhao, Xin‐Long Ma, Bin Zhao, Peng Tian, Jian‐Xiong Ma, Jia‐Yu Kang, Yang Zhang, Yue Guo, Lei Sun

**Affiliations:** ^1^ Department of Orthopaedics Tianjin Hospital Tianjin China; ^2^ Tianjin Institute of Orthopedics in Traditional Chinese and Western Medicine Tianjin China; ^3^ Department of Orthopedics Jinhua Municipal Central Hospital Jinhua Zhejiang Province China

**Keywords:** bone defect, hydroxyapatite, Naringin, silk fibroin

## Abstract

**Objectives:**

Large bone defects are a common, debilitating clinical condition that have substantial global health and economic burden. Bone tissue engineering technology has become one of the most promising approaches for regenerating defective bones. In this study, we fabricated a naringin‐inlaid composite silk fibroin/hydroxyapatite (NG/SF/HAp) scaffold to repair bone defects.

**Materials and Methods:**

The salt‐leaching technology was used to fabricate the NG/SF/HAp scaffold. The cytocompatibility of the NG/SF/HAp scaffold was assessed using scanning electron microscopy, live/dead cell staining and phalloidin staining. The osteogenic and angiogenic properties were assessed in vitro and in vivo.

**Results:**

The porous NG/SF/HAp scaffold had a well‐designed biomimetic porous structure with osteoinductive and angiogenic activities. A gene microarray identified 854 differentially expressed genes between human umbilical cord‐derived mesenchymal stem cells (hUCMSCs) cultured on SF‐nHAp scaffolds and cells cultured on NG/SF/HAp scaffolds. The underlying osteoblastic mechanism was investigated using hUCMSCs in vitro. Naringin facilitated hUCMSC ingrowth into the SF/HAp scaffold and promoted osteogenic differentiation. The osteogenic and angiogenic capabilities of cells cultured in the NG/SF/HAp scaffold were superior to those of cells cultured in the SF/HAp scaffold.

**Conclusions:**

The data indicate the potential of the SF/HAp composite scaffold incorporating naringin for bone regeneration.

## Graphical abstract

Overall schematic illustration of naringin‐inlaid silk fibroin/hydroxyapatite scaffold enhances human umbilical cord‐derived mesenchymal stem cell‐based bone regeneration.

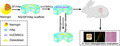

## 1 INTRODUCTION

Large bone defects caused by fracture, tumour resection and bone infection are common, debilitating clinical conditions with a substantial clinical burden.^1^, ^2^ Annually in China, over 6 million patients experience bone defects caused by trauma or various bone diseases.^3^ Autologous bone grafts, allografts and artificial bone grafts are the preferred treatments for large bone defects.^4^ Autologous bone grafts are the gold standard for treating bone defects. However, the bone supply is limited, and a second surgical site is required.^5^ Allografts are at risk of infection and immune rejection.^6^ Limitations of artificial bone grafts include osteoconductive activity, which restricts their clinical application.^7^ In general, the current treatments for large bone defects remain inadequate.^8^

Bone tissue engineering technology has emerged as a promising approach for the regeneration of defective bones.^9^, ^10^, ^11^, ^12^ Bone tissue engineering includes four elements: seed cells, growth factors, scaffolds and the culture environment.^13^ The design of engineered bone grafts requires a balance between biocompatibility and mechanical properties.^14^ Polymer‐ or organic‐based scaffolds are easily fabricated into different structures but often do not have the desired compressive modulus.^15^, ^16^, ^17^ Ceramic‐based scaffolds have a high compressive modulus but low porosity and thus display higher rates of engraftment failure than other types of scaffolds.^18^ Therefore, composite scaffolds may enhance the mechanical and biochemical properties of scaffolds and have become promising alternatives to repair and regenerate injured tissues.

Silk fibroin (SF) is a protein derived from the cocoons of the silk worm (*Bombyx mori*). SF has remarkable mechanical strength, controllable biodegradability, and excellent biocompatibility and is easy to process.^19^, ^20^ SF has been extensively used in bone tissue engineering for bone regeneration.^21^, ^22^ Composite scaffolds of SF and hydroxyapatite (HAp) fabricated using different approaches promote bone regeneration.^23^ However, the pore sizes of the resulting scaffolds are smaller and not easily controllable. The salt‐leaching method can precisely control the pore sizes of scaffolds.^24^ In a previous study, a novel SF/HAp composite scaffold was constructed by incorporating HAp into an SF‐hexafluoroisopropanol (HFIP) solution through salt‐leaching. The optimal SF:HAp:salt mass ratio was 1:1:20.^14^

Presently, we developed a nanofibrous matrix in three‐dimensional (3D) porous scaffolds using a salt‐leaching technique in conjunction with phase separation, as previously reported.^14^ Bone morphogenetic proteins (BMPs) are considered the most potent osteoinductive factors for bone tissue engineering.^25^ However, the effective application of growth factors remains challenging because of their short half‐lives, susceptibility to degradation, and potential for rapid dilution.^26^ Alternative osteogenic inducers to treat bone defects are needed. Naringin is an active flavonoid isolated from citrus fruit extracts. The variety of pharmacological activities of naringin include anti‐inflammatory, antiapoptotic, anticancer and antihypertensive activities.^27^ We previously demonstrated that naringin abrogates bone loss induced by ovariectomy,^28^ sciatic neurectomy^29^ and glucocorticoids.^30^ More importantly, we first suggested that naringin drives endothelial progenitor cell differentiation and improves angiogenesis during osteoporotic fracture healing.^31^, ^32^ Owing to the lack of vascular resources in the inner regions of thick scaffolds, seeded cells do not survive long.^33^ Naringin may also solve the problem of insufficient blood vessels in the scaffolds. In vivo and in vitro results suggest that naringin may serve as a replacement to help regenerate damaged bone tissue.^34^, ^35^ Mesenchymal stem cells (MSCs) are the most commonly used seed cells in bone tissue engineering technology, including adipose‐derived MSCs, peripheral blood‐derived MSCs, bone marrow MSCs (BMSCs), and human umbilical cord‐derived MSCs (hUCMSCs). hUCMSCs have many advantages over adipose‐derived MSCs and BMSCs, including a non‐invasive collection procedure, low risk of infection, low immunogenicity and multipotency.^36^, ^37^

To the best of our knowledge, this is the first study in which naringin was incorporated into the construction of 3D SF/HAp scaffolds. The fabricated naringin‐containing SF/HAp scaffolds were characterized using scanning electron microscopy (SEM) and Fourier transform infrared (FTIR) spectroscopy. In vitro and in vivo osteogenic differentiation assays were performed using alkaline phosphatase (ALP) staining, Alizarin red staining (ARS) and gene expression analyses. Finally, the scaffolds were used for bone defect repair in vivo.

## 2 MATERIALS AND METHODS

### 2.1 Materials

*B mori* cocoons were provided by Simatech Co., Ltd. (Suzhou, China). Hydroxyapatite was purchased from Beijing Solarbio Co., Ltd. (Beijing, China). Naringin (≥95% pure by high‐performance liquid chromatography) was acquired from Sigma‐Aldrich Co., LLC (St. Louis, MO, USA). HFIP was purchased from Aladdin, Inc (Shanghai, China). The Human Mesenchymal Stem Cell Identification Kit was purchased from TBD Science (Tianjin, China).

### 2.2 Synthesis of NG/SF/HAp scaffolds

SF/HAp scaffolds containing different concentrations of naringin (NG/SF/HAp scaffolds) were fabricated using a phase separation technique, as previously described.^14^ First, the SF solution was prepared using our previously established protocol.^19^ Briefly, small pieces of silk cocoons were degummed by boiling in a 0.02 M Na_2_CO_3_ solution for 30 min, rinsed with distilled water to remove sericin, and dried overnight. The resulting fibres were then dissolved in 9.3 M LiBr for 4 h at 60°C and then dialysed with a cellulose dialysis membrane (3500 M_w_; Solarbio) against ultrapure water for 72 h to remove the residual LiBr.

The aqueous silk solutions were lyophilized and redissolved in HFIP to yield a 16% w/v solution. Naringin is soluble in organic solvents such as dimethyl sulfoxide (DMSO) and HFIP. Accordingly, different concentrations of naringin (0.03, 0.06, and 0.1%) were blended with the silk solution. The HAp powder was mixed with NaCl particles, and the silk solution was poured over the mixture. The pore sizes of the granular NaCl ranged from 300 to 400 μm. The HAp‐silk composition was fabricated using a silk:HAp:salt mixture with a mass ratio of 1:1:20. Once the HFIP was completely volatilized, the scaffolds were treated with anhydrous ethanol for 1 d to induce β‐sheet formation. The salt was removed by immersing the scaffolds in ultrapure water for 72 h. Scaffolds were cut into 4‐mm‐diameter cylinders with a thickness of 4 mm and sterilized using cobalt‐60 irradiation before the cells were seeded.

### 2.3 Characterization of naringin/SF/HAp scaffolds

The morphology of the SF/HAp scaffolds was observed using SEM (Carl Zeiss, Oberkochen, Germany). The average size of the SF/HAp scaffolds and the pore size of the scaffolds were calculated using Nano Measure 1.2 image processing software.

The mechanical properties of the different scaffolds were assessed in triplicate after immersion in phosphate‐buffered saline (PBS) overnight. The compressive strengths of the scaffold specimens (diameter 5 mm, thickness 5 mm) were measured using the Electro‐Force 3230 System (BOSE, Minnetonka, MN, USA) at a constant loading rate of 0.5 mm/min.

The porosity of the composite scaffold was calculated based on the initial n‐hexane volume (V1), total volume of the scaffold and n‐hexane (V2), and residual n‐hexane volume (V3) using the following equation^38^:

$$\text{porosity}\;\left(\%\right)=\frac{V1‐V3}{V2‐V3}\times 100\%.$$

The absorption values at 450 nm for different concentrations of naringin was determined using a microplate fluorometer. Standard curves were drawn based on the absorption values. The 0.1 NG/SF/HAp scaffolds were immersed in 2 mL of PBS at 37°C and then shaken until the maximum naringin release was achieved. All release tests were performed in duplicate, and the experimental standard error never exceeded 15% of the experimental mean. FTIR spectra of the scaffolds were obtained using an FTIR‐7600 spectrometer (Lambda Scientific, Edwardstown, Australia).

### 2.4 Culture and identification of hUCMSCs

Ethical approval was obtained from the Ethics Committee of Tianjin Hospital (Tianjin, China). hUCMSCs were isolated and cultured as described previously.^39^ Briefly, after informed consent was provided by patients, and fresh human umbilical cords were obtained postpartum, as previously described. Umbilical cords were disinfected in 75% ethanol for 1 min, and the umbilical cord vessels were removed using ophthalmic scissors. The mesenchymal tissue (Wharton's jelly) was diced into cubes (approximately 0.5 cm) and centrifuged at 250 × g for 5 min. The mesenchymal tissue was washed with serum‐free Dulbecco's modified Eagle's medium (DMEM; Gibco, Carlsbad, CA, USA) and centrifuged (250 × g, 5 min). The supernatant was discarded. Mesenchymal tissue was treated with 0.1% collagenase (Sigma‐Aldrich) at 37°C overnight. Afterwards, tissues were centrifuged and washed with PBS twice before further digestion with 2.5% trypsin (Gibco; Thermo Fisher Scientific, Waltham, MA, USA) at 37°C for 30 min. The enzymatic reaction was stopped using DMEM containing 20% foetal bovine serum (FBS). The dissociated mesenchymal cells were further dispersed by treatment with 10% FBS‐DMEM. Cells were plated in 25‐cm^2^ culture flasks as described above.

The digested cells were washed twice with PBS and centrifuged at 300 × g for 5 min. The supernatants were discarded, and the cells were washed once in the staining buffer. The washed cells were resuspended in staining buffer at a density of 5 × 10^6^ cells/mL. Next, 100 μL of cells were placed in a flow tube, and 5 μL of blocking solution was added and incubated at room temperature for 15 min. Then, 10 μL of blocking solution, antibody combination I (anti‐human CD90 FITC, anti‐human CD105 APC, and anti‐human CD45 PE‐Cy7), antibody combination II (anti‐human HLA‐DR FITC, anti‐human CD73 APC, and anti‐human CD34 PE‐Cy7) and the isotype control were added and incubated at room temperature for 15 min in the dark. Next, 1 mL of staining buffer was added to these four flow tubes and centrifuged at 300 × g for 5 min. The supernatants were discarded, 500 μL of staining buffer was added and mixed well, and flow cytometry was performed using an FACSAria III Cellsorter (BD Biosciences, San Jose, CA, USA).

hUCMSCs were tested for their trilineage differentiation potential using Fuyuanbio differentiation kits (Fuyuan Bio, Shanghai, China) for osteogenic, adipogenic and chondrogenic differentiation according to the manufacturer’ s instructions. At the end of the induction procedure, cells that underwent osteogenic, adipogenic and chondrogenic differentiation were stained with Alizarin Red, Oil Red O and Alcian Blue solutions, respectively.

### 2.5 Cellular metabolic activity

Cell adhesion and proliferation on the NG/SF/HAp scaffolds were evaluated quantitatively at 1, 3 and 5 days after seeding. After disinfection, the surfaces of the scaffolds were seeded with hUCMSCs at a density of 1 × 10^6^ cells/mL until they reached a saturated state. The seeded scaffolds were placed in a 37°C incubator for 1 h and then transferred to another 24‐well plate, followed by addition of 1 mL of culture medium to each well. After incubation for 3 days, all scaffolds were washed twice and fixed with 4% paraformaldehyde (Solarbio) at room temperature for 24 h. The scaffolds were dehydrated and dried with a gradient of alcohol solutions for 20 min. Cell viability was assayed using a live/dead cell kit (Thermo Fisher Scientific) according to the standard protocol to verify the viability of hUCMSCs growing on different NG/SF/HAp scaffolds. After 1, 3 and 5 days of coculture, the culture medium was removed, and the cells were rinsed twice with PBS. Scaffolds were stained with live/dead staining solution (0.5 µL of calcein AM and 2 µL of ethidium homodimer‐1 diluted in 1 mL of PBS) for 10 min in the dark in an incubator. Images were acquired using a FluoView 1000 confocal microscope (Olympus, Tokyo, Japan). After 1, 3, and 5 days of cell culture, the hUCMSCs were fixed with a 4% paraformaldehyde solution (Solarbio) for 15 min at room temperature and then permeabilized with 0.5% Triton X‐100 (Solarbio) in PBS for 10 min at 4°C. After three washes with PBS, the hUCMSCs were stained with TRITC‐conjugated phalloidin (0.5 ml of 5 μg/mL, Solarbio) for 10 min, and the nuclei were counterstained blue with DAPI staining solution (Solarbio). Immunofluorescence was observed using a FluoView 1000 confocal laser scanning microscope (Olympus).

Growth kinetics of hUCMSCs on the scaffolds were evaluated using CCK‐8 assay after 1, 3 and 5 days of cell culture. At each time point, the samples were washed with 1× PBS, and 1 mL of cell medium with CCK‐8 solution (5 mg/mL stock in PBS, Sigma‐Aldrich) was added and incubated at 37°C and 5% CO_2_ for 4 h. The absorbance of the samples was monitored at 450 nm using a SpectraMax Plus 384 enzyme‐linked immunosorbent assay reader (Molecular Devices, Sunnyvale, CA, USA).

### 2.6 Osteogenic differentiation of hUCMSCs

After 7 and 28 days of induction, the cells were fixed with 4% polyformaldehyde for 15 min and assessed using ALP and ARS staining, respectively. Briefly, after fixation with polyformaldehyde, these cells were washed with PBS three times and incubated with NBT/BCIP reagent (Beyotime, Shanghai, China) and ARS solution (Solarbio) for 5 min and 30 min, respectively. The cells were photographed under a light microscope (Leica, Wetzlar, Germany).

Quantitative analysis of ALP activity was performed using an ALP (AKP/ALP) detection kit (BioVision, Milpitas, CA, USA) according to the manufacturer's instructions. Briefly, after 7 days of culture, the cells were treated with 0.5% Triton X‐100 (Solarbio) for 5 min. Total protein was obtained through a repeated freeze‐thaw process. After centrifugation at 500 × g for 10 min, the supernatants of the centrifuged samples were collected, and the absorbance was measured at 450 nm using a microplate reader (BioTek Instruments, Winooski, VT, USA).

A 10% cetylpyridinium chloride solution (TCI, Shanghai, China) was added to the wells of the plate and incubated for another 1 h to release calcium‐bound Alizarin red S and quantify mineralization. The absorbance at 562 nm was measured using a spectrophotometer and normalized to the protein content.

### 2.7 RNA sequencing

hUCMSCs (1 × 10^5^ cells/mL) in the lower chamber of a Transwell unit were cocultured with NG/SF/HAp or SF/HAp in the upper chamber of the 6‐well Transwell for 72 h to further explore the mechanism of osteogenic differentiation of the cells cultured in the NG/SF/nHAp scaffolds. After 72 h of incubation, the cells were collected, and 1 mL of TRIzol was added to extract total RNA. The experiments were performed using the Human Genome U133 Plus 2.0 Array (Affymetrix, Santa Clara, CA, USA) as described previously.^40^, ^41^ The Limma package was used to identify differentially expressed genes (DEGs). The cut‐off criteria for DEG selection were |log2‐fold change| > 1 and adjusted *P* <.01. The heatmap and Volcano plot were generated using R software. Gene Ontology (GO) analyses involved three GO categories: biological process (BP), cellular component (CC) and molecular function (MF). We also conducted Kyoto Encyclopedia of Genes and Genomes (KEGG) pathway analyses using the ‘clusterprofiler’ (version 3.3.1) in R package (version 3.6.1). The protein‐protein interaction (PPI) database STRING version 11 was used to study PPIs. Plug‐in molecular complex detection (MCODE) was used to identify highly interconnected clusters in a network. The MCODE criteria for selection were as follows: MCODE scores ≥5, degree cut‐off =2, node score cut‐off =0.2, k−core =2 and max depth =100. Gene set enrichment analysis (GSEA) was performed using Java GSEA software v2.0.13 (http://www.broadinstitute.org/gsea).

### 2.8 Quantitative reverse transcription polymerase chain reaction

Total RNA was extracted from tissues and cells using TRIzol reagent (Thermo Fisher Scientific). Total RNA was then reverse‐transcribed to cDNA using a ReverTra Ace qPCR RT Kit (Toyobo Co., Ltd., Osaka, Japan). The expression levels of target genes were assessed using quantitative reverse transcription polymerase chain reaction (qRT‐PCR) in hUCMSCs. The primers used for the qRT‐PCR are listed in Table 1. The relative quantification of the target gene levels compared with the level of the internal control gene glyceraldehyde 3‐phosphate dehydrogenase (*GAPDH*) was performed using the 2^‐ΔΔCt^ method.

**TABLE 1:** Sense and antisense primers for quantitative reverse transcription polymerase chain reaction

Genes	Forward primers (5’‐3’)	Reverse primers (5’‐3’)
*GAPDH*	GAAGGTGAAGGTCGGAGTC	GAGATGGTGATGGGATTTC
*OSX*	CCTCCTCAGCTCACCTTCTC	GTTGGGAGCCCAAATAGAAA
*RUNX2*	TCTTAGAACAAATTCTGCCCTTT	TGCTTTGGTCTTGAAATCACA
*COL1A*	GCTGATGATGCCAATGTGGTT	CCAGTCAGAGTGGCACATCTTG

### 2.9 Western blot analysis

hUCMSCs were extracted and lysed with radioimmunoprecipitation assay lysis buffer. Protein concentration was quantified using a BCA protein assay kit (Solarbio). Whole‐cell lysates containing 50 μg protein was separated using 12% sodium dodecyl sulphate‐polyacrylamide gel electrophoresis and transferred to a polyvinylidene difluoride (PVDF) membranes. The membranes were blocked with 0.1% Tris‐buffered saline containing 5% skim milk for 1 h at room temperature and then incubated overnight at 4°C with rabbit anti‐human antibodies against collagen type I A1 (COL1A1, 1:100, ab170389; Abcam, Cambridge, UK), Osteocalcin (OCN, 1:100, ab170389; Abcam), and Runt‐related transcription factor 2 (RUNX2, 1:100, ab170389; Abcam), followed by incubation with horseradish peroxidase (HRP)‐conjugated secondary antibodies (1:10,000, ab6721, Abcam) for 1 h. The immunoreactive bands were visualized using an Imager 600 (Amersham Biosciences, Buckinghamshire, UK).

### 2.10 Rabbit femoral distal bone defect

Thirty healthy 2‐month‐old male New Zealand White rabbits were obtained from the Experimental Animal Centre of Tianjin Hospital. The mean body weight was 2400 ± 320 g. The rabbits were randomly assigned to control, SF/HAp scaffold and 0.1 NG/SF/HAp scaffold groups to evaluate the osteogenic potential of the NG/SF/HAp scaffold. The rabbits were anaesthetized with intramuscular injections of 0.5 ml/kg xylazine hydrochloride (Shengda, Jilin Province, China). After the animals were anaesthetized, the skin, subcutaneous tissue and muscle were cut layer‐by‐layer. The patella and extensor mechanisms were then dissected to expose the distal aspect of the femur. Bone defects with a diameter of 6 mm and a depth of 10 mm were created by drilling in the bilateral distal aspect of the femur of each rabbit. Subsequently, the scaffolds (SF/HAp scaffold, 0.1 NG/SF/HAp) were implanted into the defects, and the wound was sutured layer‐by‐layer. No surgery was performed in the control group. Surgery was then completed with suturing and dressing of the surgical wounds. Penicillin (100 000 U/day) was injected intramuscularly each day after the operation for 3 days.

### 2.11 Microcomputed tomography

At 4 weeks post‐implantation, the rabbit femoral condyle was obtained and fixed with 4% paraformaldehyde. The tissues of the rabbit femoral condyle defect repair site were analysed using microcomputed tomography (μCT) (Siemens, Berlin, Germany). After scanning, 3D reconstruction was performed automatically. The following scaffold parameters were also compared in this study: bone volume (BV, mm^3^)/total volume (TV, mm^3^), trabecular thickness (TB.TH), trabecular separation (TB.SP), bone surface (BS)/BV and bone mineral density (BMD, mg/cm^3^). Bone formation and mineral apposition rates (MAR) were evaluated histomorphometrically by quantifying the fluorescence of calcein (green) and tetracycline (red).

### 2.12 Histological analysis

The distal femurs at 4 weeks post‐implantation were harvested and fixed with 4% paraformaldehyde for 48 h. Hearts, livers and kidneys of each group were harvested at 4 and 8 weeks post‐implantation and fixed with 4% paraformaldehyde for 48 h. Distal femurs were decalcified in a 15% (w/v) disodium ethylenediaminetetraacetate dihydrate (EDTA) solution for 6 weeks at room temperature. After dehydration through an alcohol gradient, the samples were embedded in paraffin blocks, and 5‐mm‐thick sections were cut at the centre of the specimens. Serial sections were stained with haematoxylin and eosin (HE), toluidine blue and safranin O.

The number of osteoblasts in each group was normalized to the bone surface and quantified using ImageJ software (National Institutes of Health, Bethesda, MD, USA).

The sections were also used for immunohistochemical staining of COL1A1 and CD31. Endogenous peroxidase activity was quenched by treatment with 3% H_2_O_2_ for 10 min. The tissue sections were then boiled in citric acid buffer (10 mM citric acid) for 10 min for antigen retrieval and then incubated with primary antibodies against COL1A1 (mouse anti‐rabbit, 1:100; Santa Cruz Biotechnology, Santa Cruz, CA, USA) or CD31 (mouse anti‐rabbit, 10 μg/mL; Abcam) at 4°C overnight. Streptavidin‐biotin complex (SABC) and 3,3'‐diaminobenzidine tetrahydrochloride (DAB) visualization was performed according to the manufacturer's instructions (Servicebio Company, Wuhan, China). Nuclear counterstaining was performed using haematoxylin. Images were captured using a microscope (Nikon, Tokyo, Japan).

### 2.13 Statistical analyses

All results are presented as the mean ±standard deviation and were compared using SPSS software (version 22.0; IBM SPSS, Armonk, NY, USA). Paired or unpaired t tests were applied to compare differences between two groups. One‐way or two‐way analysis of variance (ANOVA) along with Tukey's multiple comparisons test was used to compare multiple groups. Statistical significance was set at *P* <.05.

## 3 RESULTS

### 3.1 Characterization of as‐fabricated scaffolds

**FIGURE 1:**
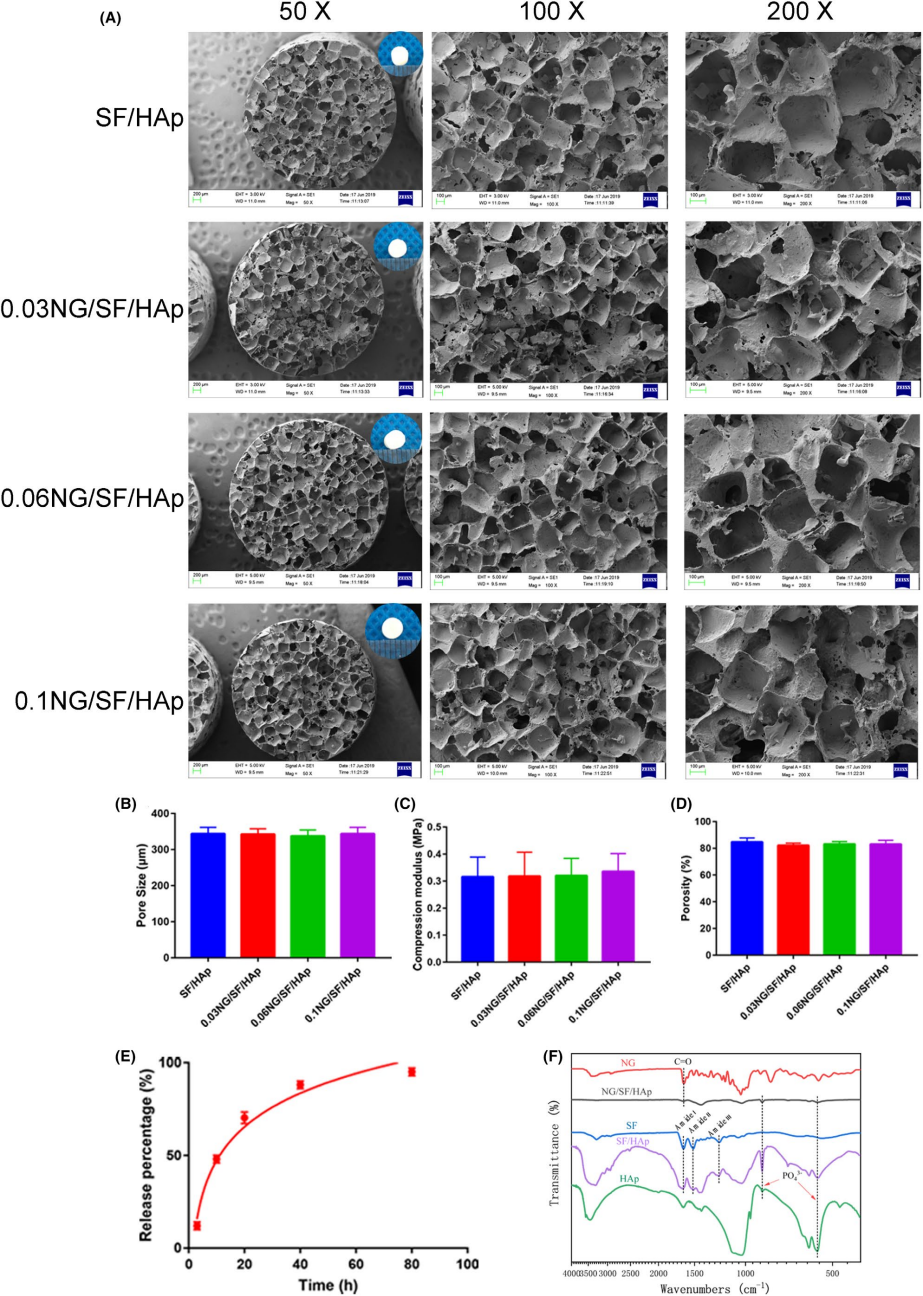
Fabrication and characterization of the SF/HAp scaffolds in vitro. (A) Typical SEM images of the SF/HAp and SF/HAp scaffolds inlaid with different concentrations of naringin. (B) Pore size, (C) compressive strength, and (D) porosity of the SF/HAp and SF/HAp scaffolds inlaid with different concentrations of naringin. (E) Summarized data showing the in vitro naringin release rate from 0.1 NG/SF/HAp scaffolds at different time points. (F) FTIR spectra of NG/SF/HAp scaffolds

Figure 1A shows typical SEM images of the SF/HAp scaffold and SF/HAp scaffolds inlaid with different wt% (0.03, 0.06 and 0.1%) of naringin. A representative pure SF scaffold is shown in Figure S1.

All four scaffolds displayed similar shapes in response to salt treatment. The distribution of nHAp was relatively uniform, and no clusters of nHAp were observed. The mean pore diameters of SF/HAp, 0.03 NG/SF/HAp, 0.06 NG/SF/HAp and 0.1 NG/SF/HAp were 343.9 μm, 342.4 μm, 337.5 μm and 343.8 μm, respectively (Figure 1B). No statistically significant differences were observed between the pore diameters of the four scaffolds. Figure 1C shows the mechanical characteristics of the as‐synthesized SF/HAp and NG/SF/HAp scaffolds. The measured compressive property was 0.316 ± 0.07 MPa, 0.318 ± 0.09 MPa, 0.320 ± 0.06 MPa and 0.336 ± 0.07 MPa for the as‐fabricated SF/HAp, 0.03 NG/SF/HAp, 0.05 NG/SF/HAp and 0.1 NG/SF/HAp scaffolds, respectively. Significant differences were not observed in the measured compressive properties between the SF/HAp scaffold and SF/HAp scaffolds inlaid with different concentrations of naringin (F = 0.077, *P* >.05).

The porosity of SF/HAp, 0.03 NG/SF/HAp, 0.06 NG/SF/HAp and 0.1 NG/SF/HAp was 84.7 ± 3.02%, 82.2 ± 1.69%, 83.2 ± 1.87% and 83.1 ± 2.88%, respectively (Figure 1D).

The 0.1 NG/SF/HAp scaffold released 70% of the naringin within the first 20 h with an initial fast‐release profile followed by a relatively slow release (Figure 1E). The FTIR spectra of pure SF, HAp, SF/HAp and 0.1 NG/SF/HAp scaffolds are shown in Figure 1F. The FTIR spectrum of SF showed typical characteristic peaks of amide‐I at 1653 cm^‐1^, amide‐II at 1531 cm^‐1^, and amide‐III at 1235 cm^‐1^ and the spectrum of HAp displayed typical characteristic peaks at 1026 cm^‐1^ and 750‐500 cm^‐1^. In the naringin spectrum, the bands at 1080 cm^‐1^ and 1641 cm^‐1^ were assigned to the peaks corresponding to C–O and C = O, respectively. The FTIR spectra of the SF/HAp and NG/SF/HAp scaffolds further confirmed the assembly of HAp into the SF scaffold based on the presence of characteristic peaks for silk, P–O and naringin.

SF/HAp scaffolds with different concentrations of naringin degraded over time. At 8 weeks, the scaffold volume had decreased to approximately 50%, but the weight had not changed significantly (Figure S2).

### 3.2 Identification of hUCMSCs

**FIGURE 2:**
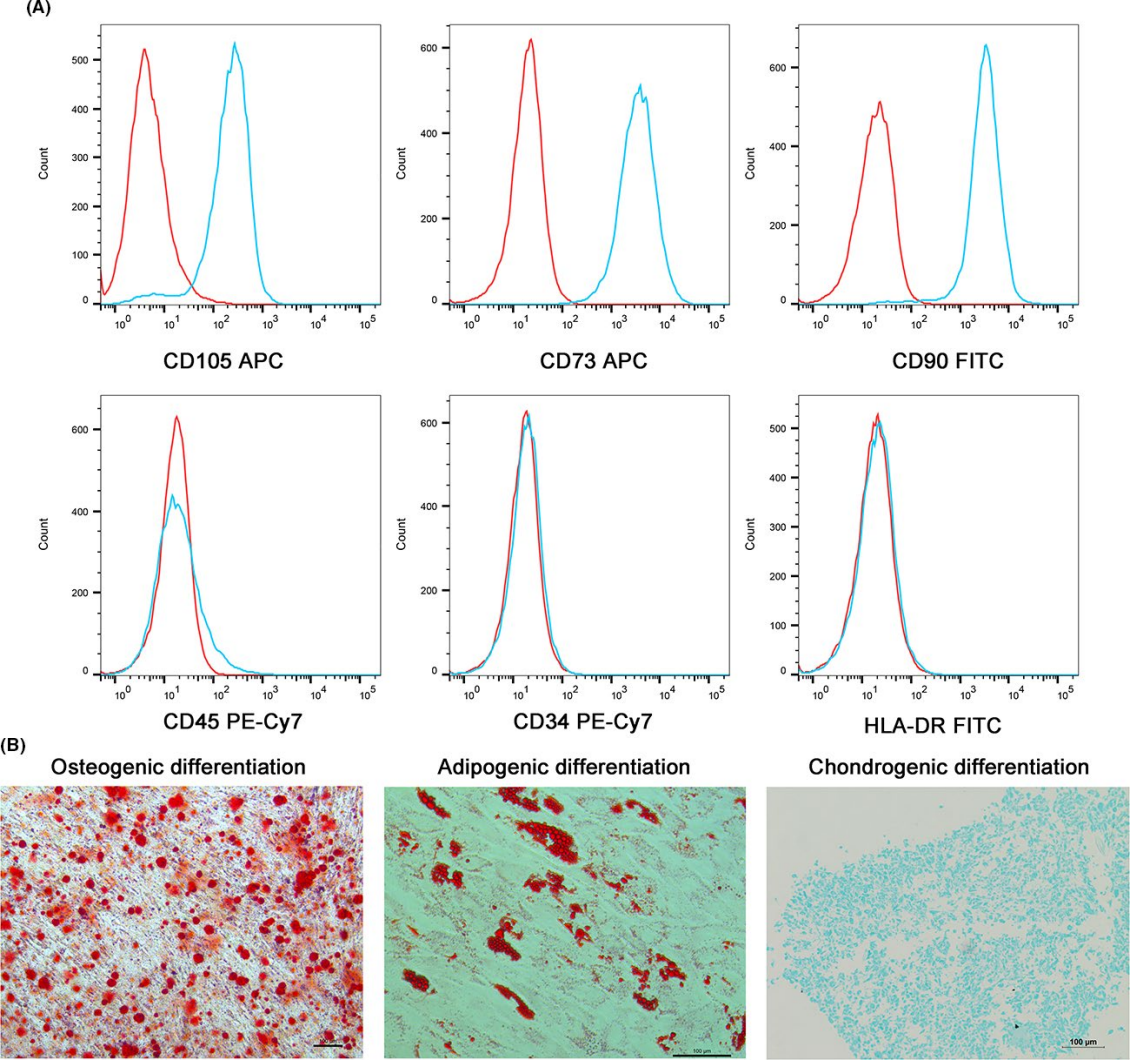
Phenotypic identification and trilineage differentiation potential of hUCMSCs. (A) hUCMSCs were negative for CD45, CD34 and HLA‐DR expression and positive for CD105, CD73 and CD90 expression. (B) hUCMSCs were cultured and induced to undergo trilineage differentiation: osteogenic differentiation, adipogenic differentiation and chondrogenic differentiation

To characterize the cultured hUCMSCs, flow cytometry was used to detect the phenotypes (CD105, CD73, CD90, CD45, CD34 and HLA‐DR) and characteristics of the cultured hUCMSCs. Trilineage differentiation potential assays were performed to determine their differentiation potential. The isolated hUCMSCs were negative for CD45, CD34, and HLA‐DR and positive for CD105, CD73 and CD90 (Figure 2A). In addition, these cells successfully differentiated into osteoblasts, adipocytes and chondroblasts (Figure 2B). The isolated cells met the International Society for Cellular Therapy position statement for hUCMSC identification.^42^

### 3.3 Effects of naringin on hUCMSC morphology and proliferation

**FIGURE 3:**
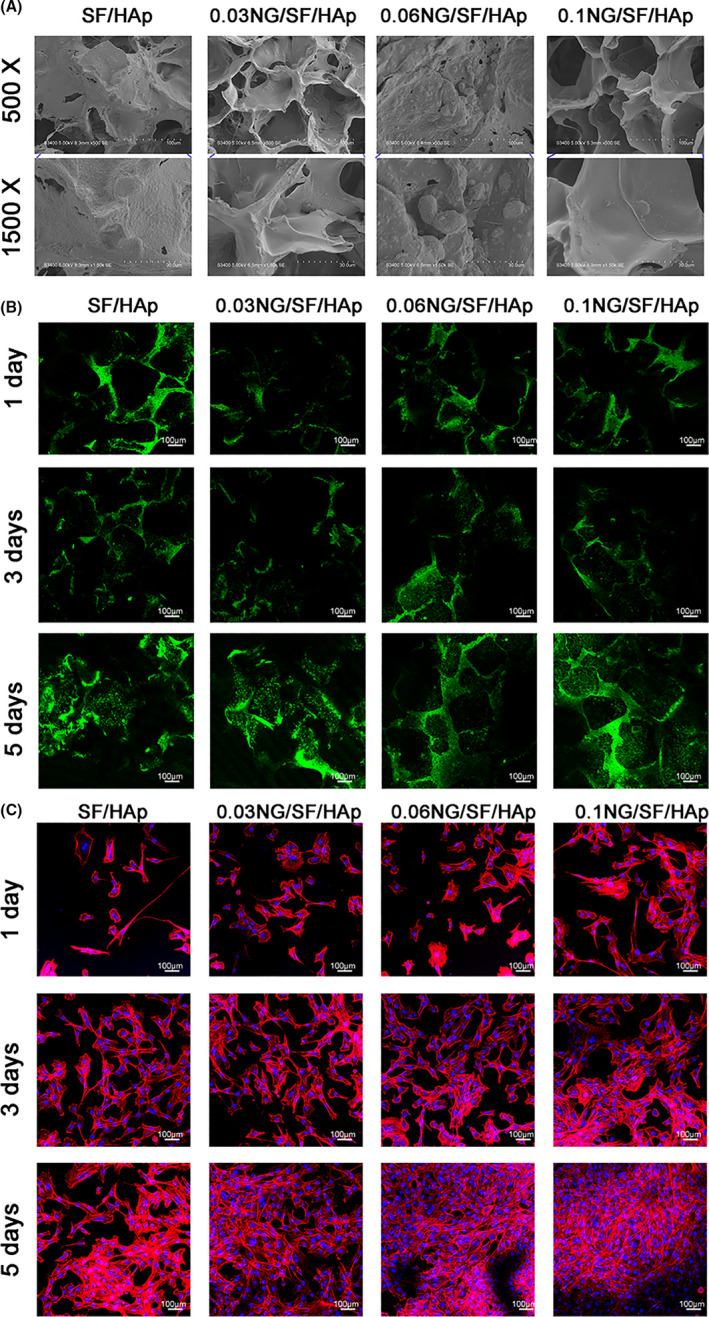
In vitro biocompatibility of hUCMSCs with different scaffolds. (A) Morphology of hUCMSCs grown on different scaffolds was observed using SEM. (B) Live (green)/dead (red) cell staining of hUCMSCs grown on SF/HAp and NG/SF/HAp scaffolds at 1, 3 and 5 days after plating. (C) hUCMSCs were stained with rhodamine‐conjugated phalloidin and DAPI on days 1, 3 and 5 of cell culture

The effects of naringin on hUCMSC adhesion on scaffolds were assessed using SEM. As shown in Figure 3A, the hUCMSCs displayed good adhesion to all scaffolds. The hUCMSCs grown on the NG/SF/HAp scaffolds displayed a spindle and osteoblastic‐like morphology compared with cells grown on the SF/HAp scaffold. Cell viability was evaluated after the cells were implanted into the scaffolds. The cells cultured for 1, 3 and 5 days were stained with a live/dead cell staining kit (Figure 3B). The number of cells on the scaffold increased over time in all the scaffolds. A few dead cells were observed on the scaffolds at 1, 3 and 5 days, indicating that the cells grown on the scaffolds remained viable.

Cell adhesion was determined by staining cells with phalloidin to label actin fibres (red fluorescence) and with a nuclear stain (blue fluorescence) on days 1, 3 and 7 after seeding (Figure 3C). The hUCMSCs grew robustly and showed good stretching on all four scaffolds, suggesting that the scaffolds were suitable for cell attachment. A cytotoxicity assay with a CCK‐8 kit revealed no significant difference in absorbance between the leaching fluids of the different scaffolds at day 0 and day 1 (Figure S3). After 3 days, the proliferation ability of hUCMSCs in the 0.06 NG/SF/HAp and 0.1 NG/SF/HAp scaffold groups was stronger than that in the SF/HAp and 0.03 NG/SF/HAp groups. After 5 days, the proliferation ability of hUCMSCs in the 0.03 NG/SF/HAp, 0.06 NG/SF/HAp and 0.1 NG/SF/HAp scaffold groups was stronger than that in the SF/HAp group (*P* <.05).

### 3.4 hUCMSC biocompatibility on SF/HAp and NG/SF/HAp scaffolds

**FIGURE 4:**
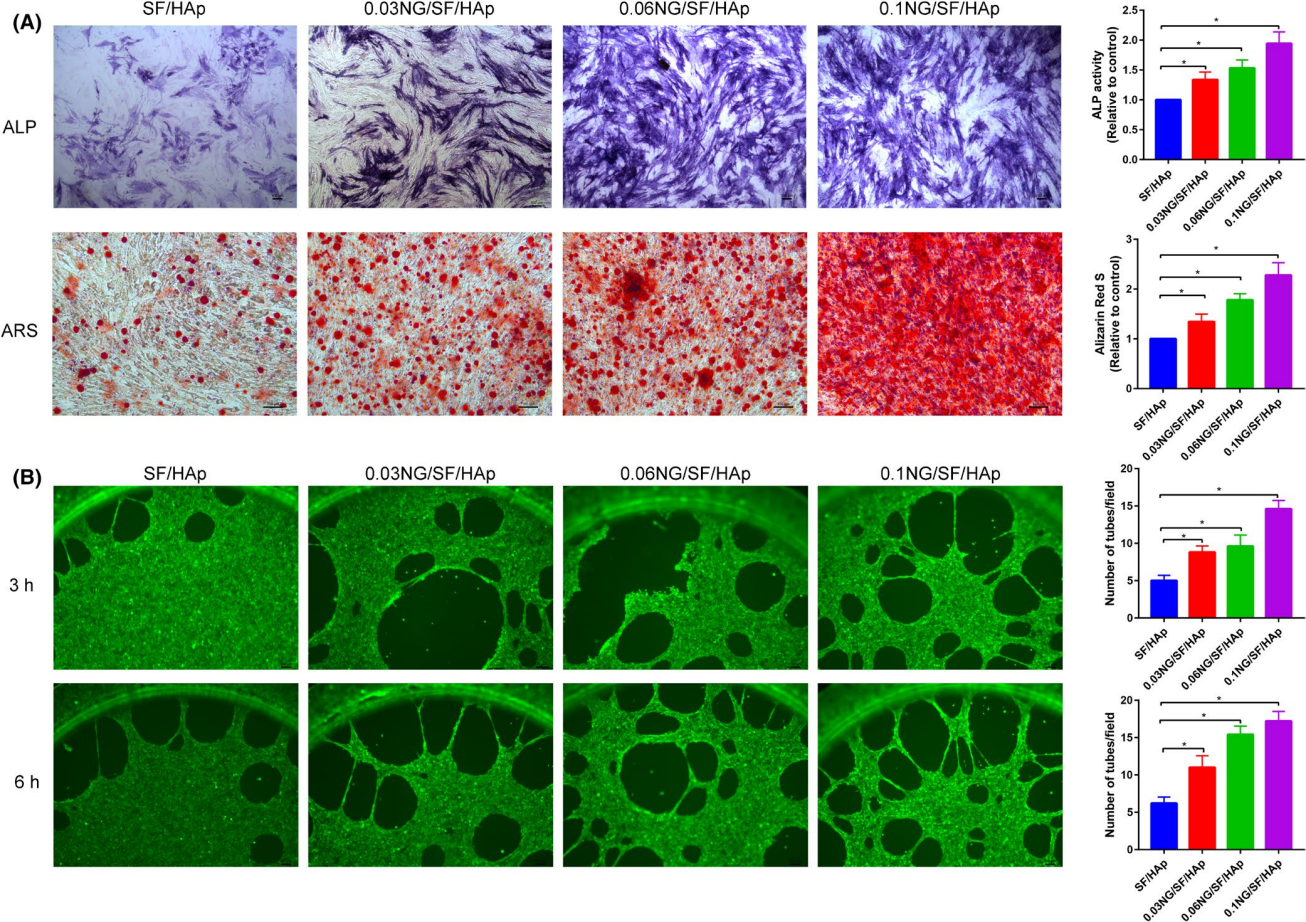
Effects of different scaffolds on the osteogenic capacity of hUCMSCs and angiogenic capacity of HUVECs. A, Representative digital images of ALP staining and Alizarin red S staining showing the early and late osteogenic capacity of hUCMSCs at 7 and 21 days of osteogenic induction, respectively. B, Microscopy images of tube formation by HUVECs after culture with different scaffold media on Matrigel for 3 and 6 h at 37°C

The abilities of SF/HAp and NG/SF/HAp to promote osteogenesis were assessed. The ALP activities and calcium contents of the hUCMSC cultures with scaffolds incorporated with different wt% naringin were investigated. The results are presented in Figure 4A. The ALP activity was significantly higher in cells grown on NG/SF/HAp scaffolds than in cells grown on SF/HAp scaffolds.

In addition, tube formation assays indicated that 0.1 NG/SF/HAp significantly increased the tube formation ability of human umbilical vein endothelial cells (HUVECs) (Figure 4B) at 3 h and 6 h, suggesting that 0.1 NG/SF/HAp promoted endothelial cell growth and activated vascularization.

### 3.5 RNA sequencing

**FIGURE 5:**
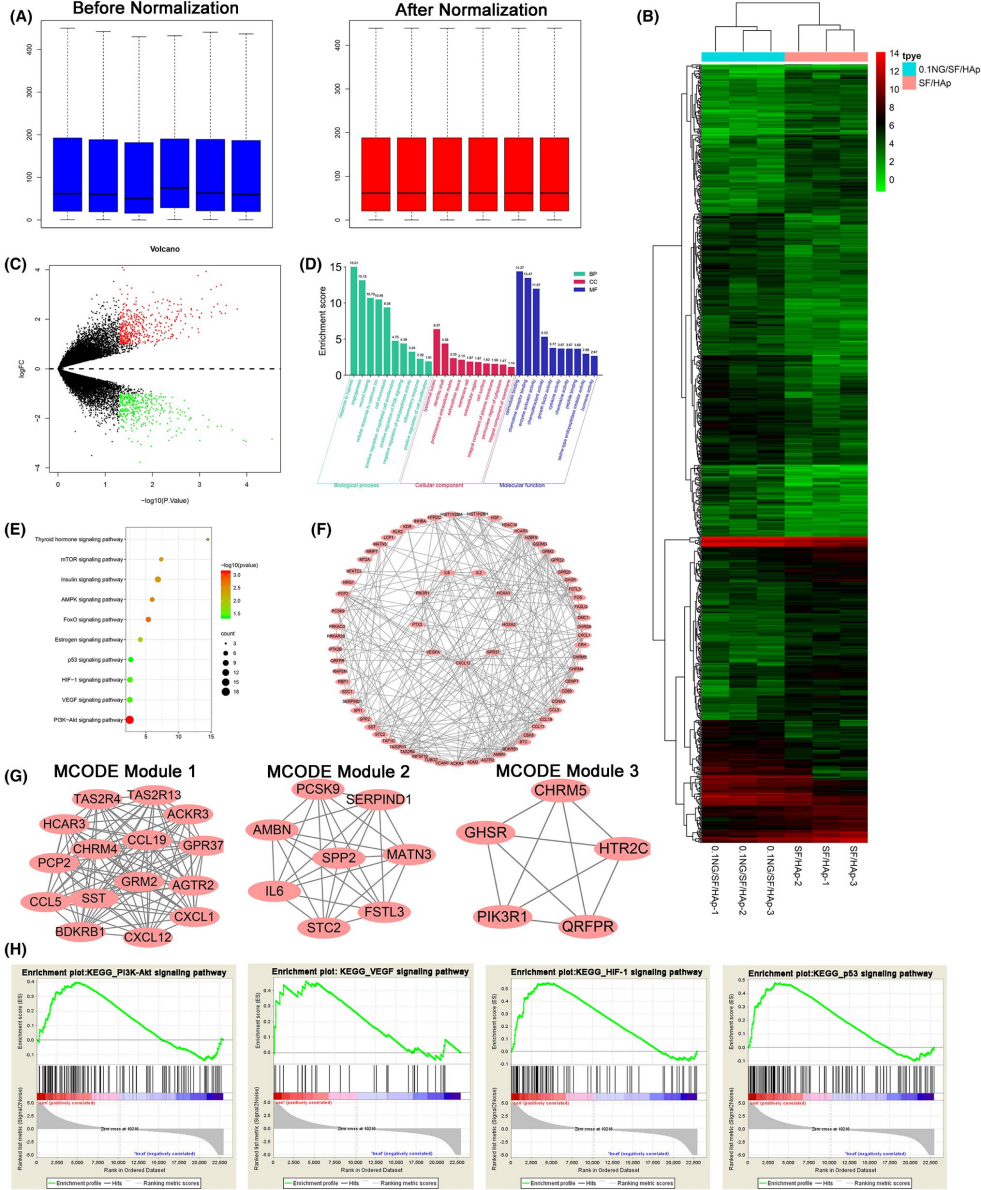
Cluster analysis of genes expressed in hUCMSCs grown on SF‐HAp and 0.1 NG/SF/HAp scaffolds. (A) Boxplots of log expression values before and after normalization. (B) Clustering heatmap of DEGs in hUCMSCs grown on the SF/HAp and 0.1 NG/SF/HAp scaffolds. (C) Differentially downregulated (green spots) and upregulated (red spots) genes expressed in SF/HAp‐ versus 0.1 NG/SF/HAp‐treated hUCMSCs were identified using a Volcano plot. (D) GO functional classification of the DEGs. The distributions are summarized in three main categories: BP, CC and MF. The x‐axis indicates different GO terms, and the y‐axis indicates the enrichment score in each category. (E) Scatter plot of enriched KEGG pathways and statistics. The colour and size of the dots represent the ‐log10 (p value) and number of genes mapped to the indicated pathways, respectively. (F) PPI networks of DEGs. (G) Module 1, MCODE score =7.564.182; module 2, MCODE score =6.287; and module 3, MCODE score =5.507. (H) Pathway analysis of the four clusters using GSEA

A box plot of the log expression values for all genes in each sample before and after normalization was plotted (Figure 5A). The median values of the samples were nearly identical, indicating that the data should be further analysed. Eight hundred fifty‐four DEGs were identified. Of these, 392 were downregulated and 462 were upregulated. The heatmap and Volcano plot of the DEGs between SF/HAp and 0.1 NG/SF/HAp are shown in Figure 5B and C, respectively. GO analysis of these DEGs is shown in Figure 5D. The main GO BPs that were enriched were the response to hypoxia, angiogenesis, wound healing, cellular response to cadmium ions, cell chemotaxis, positive regulation of epithelial cell proliferation, positive regulation of phosphoinositide 3‐kinase (PI3K) signalling, negative regulation of endopeptidase activity, inflammatory response and positive regulation of cell proliferation.

The main CCs that were enriched included the lysosomal lumen, dendritic shaft, proteinaceous extracellular matrix, extracellular space, membrane raft, extracellular region, cell surface, integral component of plasma membrane, perinuclear region of the cytoplasm and integral component of the membrane. Calmodulin binding, chemokine receptor binding, enzyme activator activity, chemoattractant activity, growth factor activity, cytokine activity, chemokine binding, peptide binding, serine‐type endopeptidase inhibitor activity and hormone activity were the main enriched MFs.

The main enriched KEGG pathways were the PI3K/Akt, vascular endothelial growth factor (VEGF), hypoxia‐inducible factor‐1 (HIF‐1), p53, oestrogen, FoxO, AMP‐activated protein kinase, insulin, mammalian target of rapamycin (mTOR) and thyroid hormone signalling pathways (Figure 5E).

Using the STRING online database and Cytoscape software, 69 DEGs were filtered into the DEG PPI network, which contained 75 nodes and 165 edges (Figure 5F). Three significant modules were constructed from the PPI network of the DEGs using MCODE: module 1 (MCODE score =31.78), module 2 (MCODE score =18.78) and module 3 (MCODE score =10.34) (Figure 5G). The results of GSEA suggested the upregulation of the ‘PI3K‐Akt signalling pathway’, ‘VEGF signalling pathway’, ‘HIF‐1 signalling pathway’ and ‘p53 signalling pathway’, indicating that the results of the enrichment analyses of DEGs were reliable (Figure 5H).

### 3.6 Bone‐specific gene expression

**FIGURE 6:**
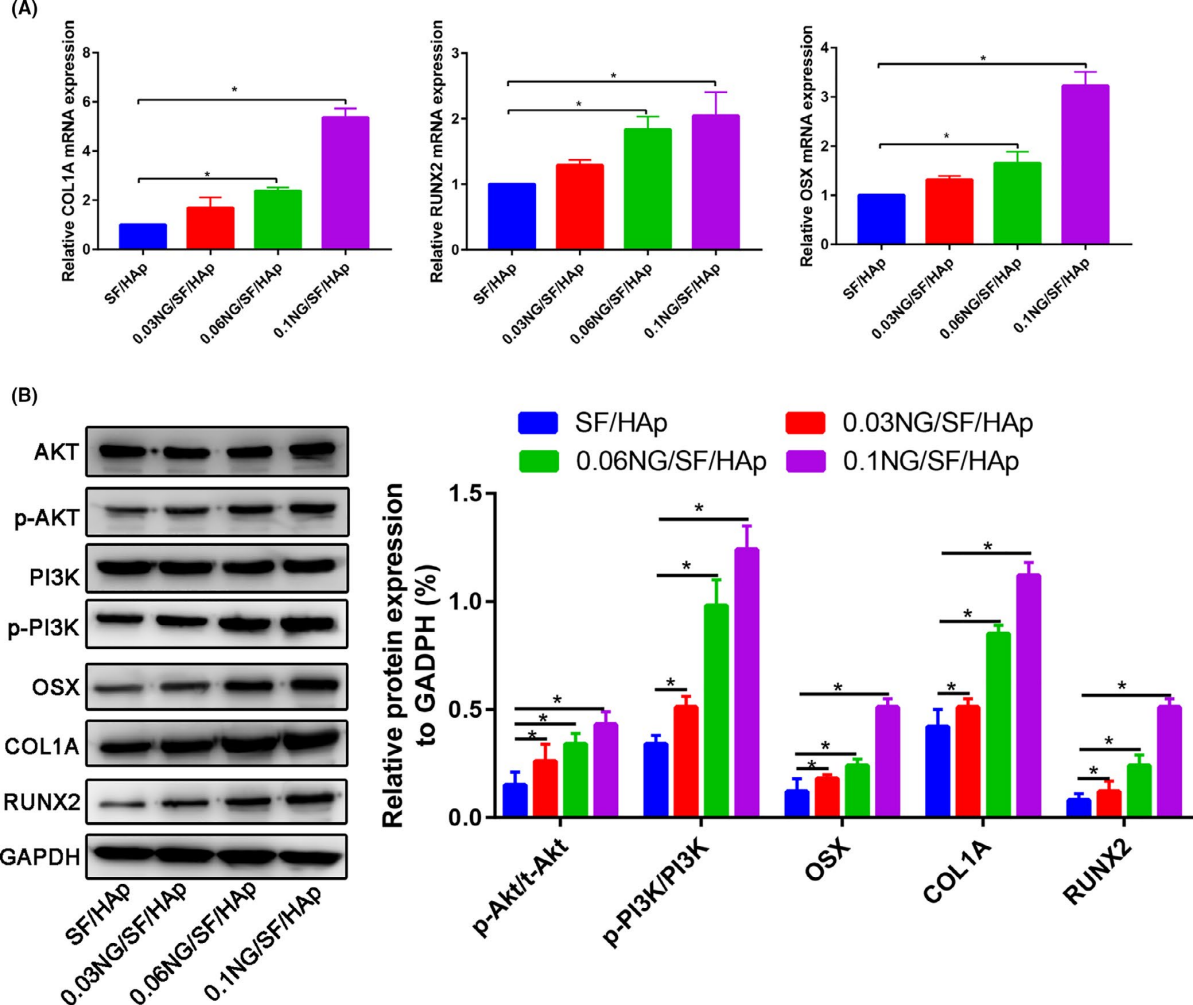
Effects of the NG/SF/HAp scaffold on osteogenesis‐related gene and protein expression in hUCMSCs cultured in scaffolds (SF/HAp and 0.1 NG/SF/HAp). (A) RT‐PCR assay. (B) Levels of osteogenesis‐related proteins and proteins involved in the PI3K/Akt pathway were analysed using western blotting

After culturing hUCMSCs in SF/HAp and NG/SF/HAp scaffolds for 14 days, the gene expression levels of osteogenic markers, including RUNX2, osterix (OSX) and COL1A1, were assessed in hUCMSCs using qRT‐PCR. Compared with the SF/HAp group, the 0.1 NG/SF/HAp group displayed upregulation of *RUNX2*, *OSX* and *COL1A* by 2.043‐, 5.360‐ and 3.228‐fold, respectively (Figure 6A).

Next, we evaluated the levels of bone formation‐related proteins using western blot analysis. The effects of NG/SF/HAp on osteogenesis‐related protein and gene expression were consistent (Figure 6B). The possible involvement of phosphorylated (p)‐PI3K and p‐Akt was examined to further elucidate the molecular mechanisms underlying the role of NG/SF/HAp in promoting osteogenesis. NG/SF/HAp had no effect on PI3K and Akt expression but strongly promoted PI3K phosphorylation (Figure 6B), indicating that NG/SF/HAp increased PI3K and Akt activity in hUCMSCs.

### 3.7 μCT

**FIGURE 7:**
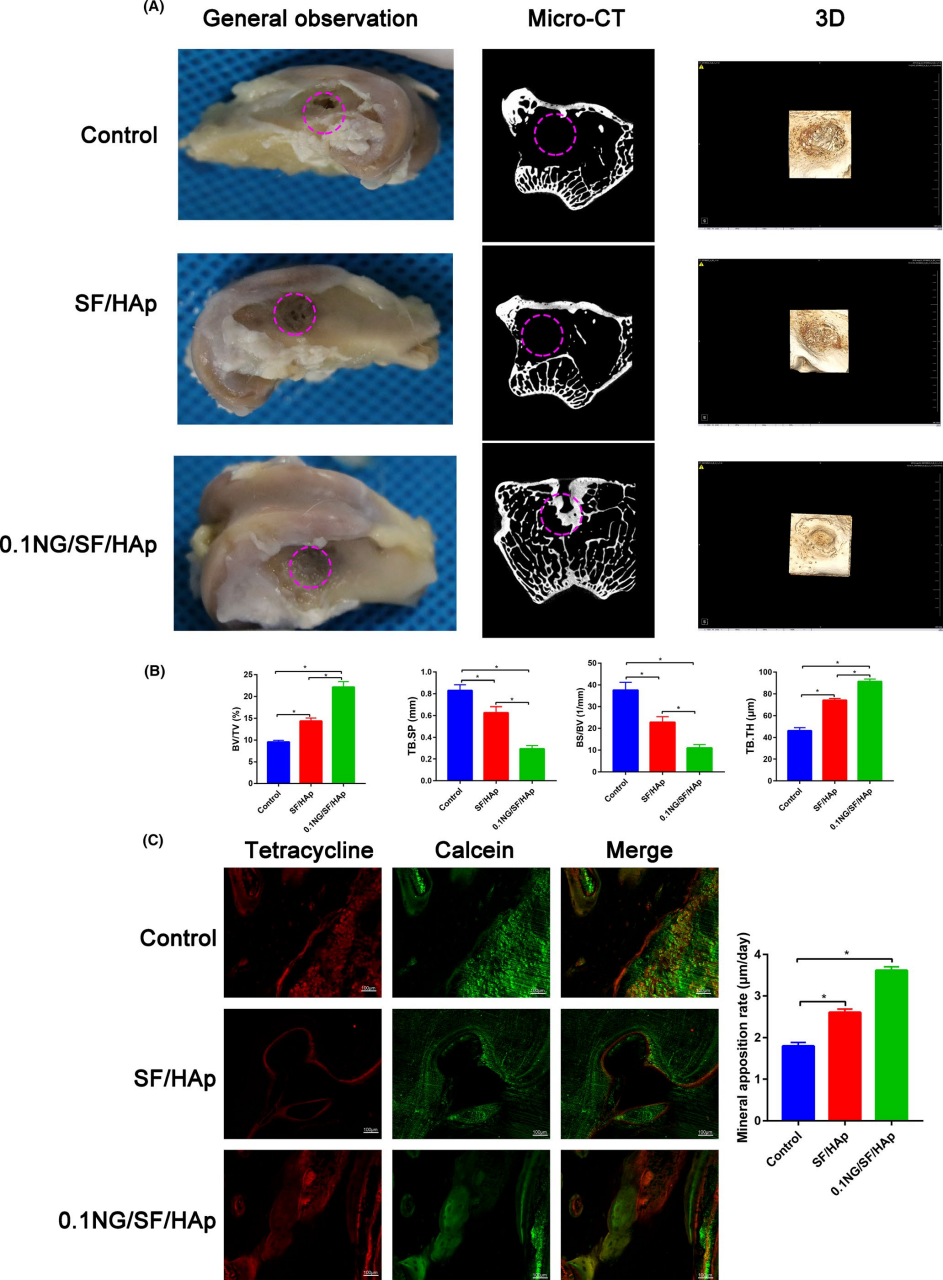
µCT analysis of new bone tissues formed in scaffolds at 4 weeks after implantation. (A) General observation and representative two‐dimensional and three‐dimensional reconstructed µCT images of SF/HAp and 0.1 NG/SF/HAp scaffolds on new bone tissue formation inside the defect site. Pink circles represent the bone defect area. (B) Summarized data showing the BV/TV, TB.SP, BS/BV and TB.TH in rabbits treated with the control, SF/Hap, and 0.1 NG/SF/HAp scaffolds (* *P* <.05). (C) Representative images of tetracycline and calcein labelling at 4 weeks after scaffold implantation

The gross appearance of the bone defects at 8 post‐operative weeks was observed. In the images, the pink dotted circles indicate the defect sites. Regenerated bone was detected in the bone defects treated with the 0.1 NG/SF/HAp scaffolds, whereas the control groups displayed little regenerated bone in the bone defects (Figure 7A). To assess bone regeneration, we performed µCT analysis 4 weeks after the creation of bone defects in the distal femur of rabbits. Bone defects persisted in the control group and little new bone tissue was observed, whereas the defect site implanted with the 0.1 NG/SF/HAp scaffolds showed bone tissue filling and mineralization at 4 weeks after surgery (Figure 7A). The BV/TV and TB.TH of the 0.1 NG/SF/HAp scaffold group were significantly higher than those of the SF/HAp scaffold and control groups at 4 weeks post‐surgery. The opposite results were observed for TB.SP and BS/TV (Figure 7B). The 0.1 NG/SF/HAp group displayed an obvious increase in MAR compared with that of the SF/HAp and control groups (Figure 7C).

### 3.8 Histological observation

**FIGURE 8:**
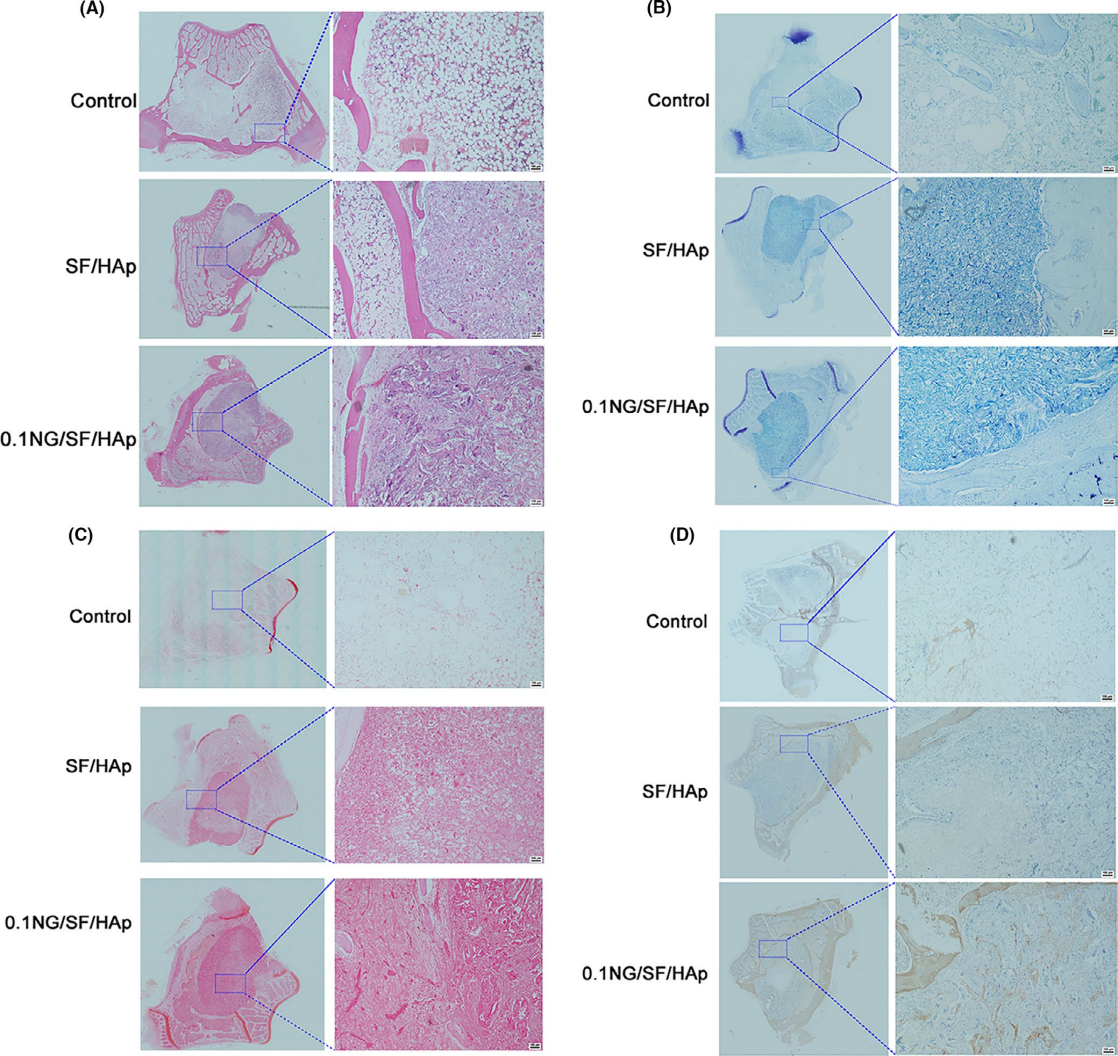
Histological analysis of defect sites at 4 weeks after surgery. (A) HE, (B) toluidine blue, (C) safranin O and (D) immunohistochemical staining for COL I in scaffold constructs at 4 weeks post‐implantation in vivo

Newly formed bone and fibrous connective tissues were stained with HE and toluidine blue, respectively, to investigate the remodelled tissue within the bone defect area. HE staining did not show obvious inflammatory reactions in any of the groups (Figure 8A). In the control group, only several scattered fibrous tissues were observed in the bone defect area. The SF/HAp or 0.1 NG/SF/HAp scaffolds completely filled the defect area and space, and fibrous connective tissues were observed among the scaffolds.

The osteoblast number per millimeter of the bone surface was quantified by manually counting osteoblasts. The results shown in Figure S4 suggested that the osteoblast number in 0.1 NG/SF/HAp was higher than that in the SF/HAp and control groups.

Toluidine blue staining revealed a line between the host bone and the native bone, and more new bone was formed in the 0.1 NG/SF/HAp group than in the other groups (Figure 8B). Safranin O staining showed that the defects in the control group were filled with only a little fibrous tissue and few chondrocytes. No bridging bone formation was observed at the defect site (Figure 8C). Chondrocyte aggregation and glycosaminoglycan deposition were observed in the SF/HAp and 0.1 NG/SF/HAp scaffolds. Immunohistochemical staining for collagen type I (COL I) showed new bone formation, with the positively stained area appearing brown in colour. Immunohistochemical staining for COL I showed higher expression in the group treated with 0.1 NG/SF/HAp scaffolds than in those in the SF/HAp and control groups (Figure 8D). Immunohistochemical staining for CD31 revealed higher expression of CD31 in the group treated with 0.1 NG/SF/HAp scaffolds than in the SF/HAp and control groups (Figure S5).

### 3.9 In vivo biosecurity of the NG/SF/HAp scaffold

**FIGURE 9:**
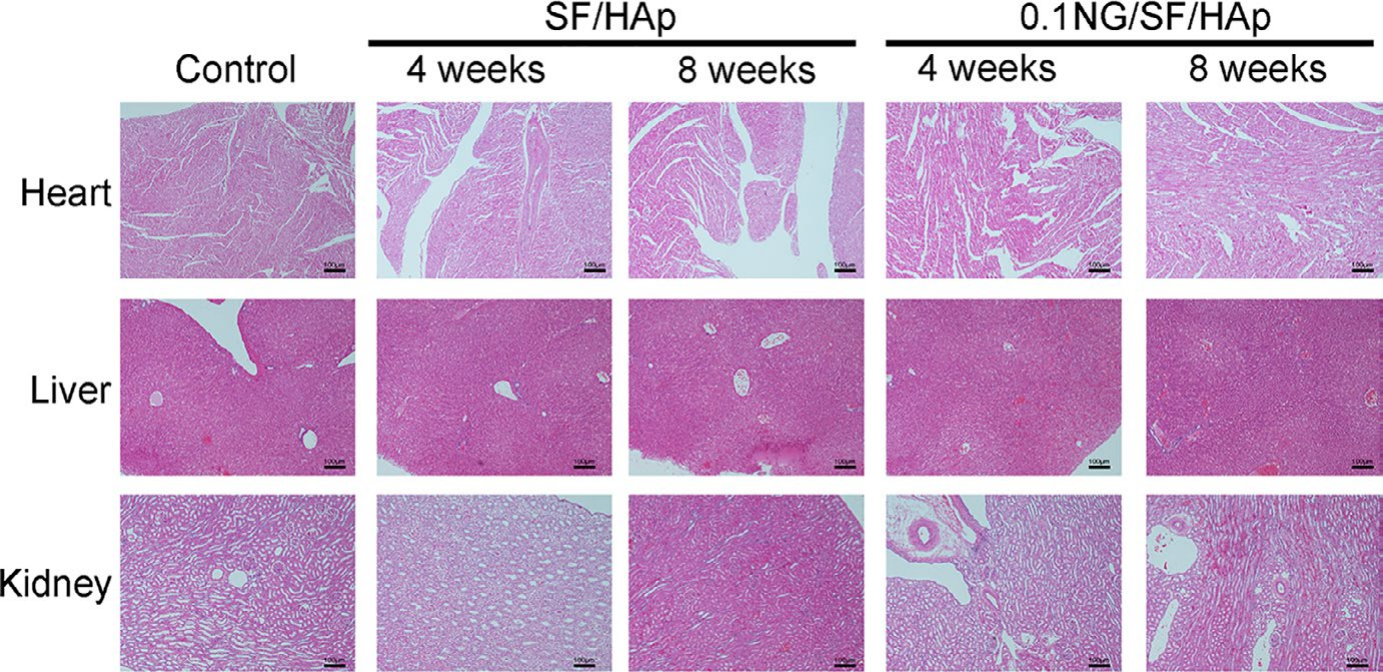
Systemic toxicity to the heart, kidney and liver of the host animals implanted with SF/HAp and 0.1 NG/SF/HAp at 4 and 8 weeks

Next, we assessed the biosecurity of the SF/HAp and 0.1 NG/SF/HAp scaffolds. HE staining of the myocardium, liver and kidney did not reveal apparent pathological changes in any of the three groups (Figure 9).

## 4 DISCUSSION

Using the salt‐leaching technology, we constructed a potentially biocompatible and osteoinductive NG/SF/HAp composite scaffold. Sustained naringin release was detected for up to 80 h. The microstructure of the NG/SF/HAp scaffold was porous, interconnected and displayed good histocompatibility for cell ingrowth and stretching. Moreover, the NG/SF/HAp scaffold exhibited osteogenic and angiogenic differentiation potential and is a promising candidate for tissue engineering. Finally, results indicated that NG/SF/HAp primarily functions by regulating the activation of the PI3K/Akt signalling pathway.

Different concentrations of naringin were incorporated into SF/HAp scaffolds to efficiently fabricate bone tissue‐engineered grafts. Salt‐leaching was used to fabricate the NG/SF/HAp scaffolds. SF, HAp and naringin were readily soluble in HFIP. We determined the SF and HAp mass ratio of 20:1 according to a previous study.^14^ The hydroxyl groups in naringin were immobilized by the carboxylic end groups of SF and HAp through chemical bonding. For this reason, naringin was released very slowly, and 90% of the drug was released after 80 h. The naringin phytomolecule enhances osteogenesis and mineralization.^43^, ^44^, ^45^, ^46^ Our group has demonstrated that naringin prevents bone loss induced by sciatic neurectomy,^29^ ovariectomy^28^ and glucocorticoids.^30^ Moreover, naringin promoted fracture healing by stimulating angiogenesis in osteoporotic rats and stimulated tube formation in endothelial progenitor cells.^31^, ^32^ However, naringin easily loses its bioactivity when directly immersed in a fluid environment. The incorporation of naringin into the SF/HAp scaffold maintained the biological activity of naringin. The pore sizes of the NG/SF/HAp scaffolds exceeded 300 μm, and the porosity was greater than 80%. These characteristics favour the use of NG/SF/HAp scaffolds as bone graft substitutes. The incorporation of naringin into the SF/HAp scaffold did not alter the pore size, porosity or mechanical properties of the natural cancellous bone‐like structure of the scaffold.

We explored the cytotoxic effects of NG/SF/HAp on hUCMSCs. The morphology of the attached cells and live/dead cell staining indicated that NG/SF/HAp had no cytotoxic effects and was suitable for cell growth. NG/SF/HAp promoted the osteogenic differentiation of hUCMSCs and angiogenesis of HUVECs in vitro. We then utilized a global gene microarray assay to better understand the underlying mechanisms of the osteoinductivity of NG/SF/HAp. A previous study compared DEGs between SF/HAp and SF scaffolds.^47^ This study revealed that HAp mainly enhanced MSC‐based bone regeneration via the interleukin‐1 alpha autocrine/paracrine signalling loop. In addition to this signalling pathway, other studies suggested that the HAp osteoinductive mechanisms were mainly related to bone development pathways such as the BMP/Smad,^48^, ^49^ Wnt, transforming growth factor‐beta,^50^ mitogen‐activated protein kinase and Notch signalling pathways.^49^ Based on the gene microarray assay results, the mechanism of action of naringin differs from the reported mechanism. NG/SF/HAp mainly activated the PI3K/Akt, VEGF and HIF‐1 signalling pathways. Previously, we showed that naringin regulated the PI3K/Akt and VEGF pathways.^31^, ^32^ The HIF‐1 signalling pathway is upstream of PI3K and VEGF signalling. Future studies should focus on changes in HIF‐1 levels after naringin treatment.

SF/HAp scaffolds with and without naringin were used for in vivo studies for 4 weeks. Enhanced bone tissue formation was evident in rabbit distal femur defect models. µCT and histology analyses suggested that the 0.1 NG/SF/HAp scaffold enhanced bone formation at the site of reconstruction in rabbit distal femur defects. Moreover, 0.1 NG/SF/HAp possessed a faster MAR within the bone defect region at 4 weeks after scaffold implantation than that of the control and SF/HAp groups.

Angiogenesis and neovascularization are critical processes in the repair of large bone defects. Previously, the failure of implants to heal large bone defects was often attributed to insufficient bone healing, mainly due to insufficient vascularization.^51^ The formation of a functional vascular system is critical for the delivery of nutrients, removal of byproducts and gas exchange. Previously, Kandhare et al^52^ revealed that naringin promoted angiogenesis and inhibited endothelial apoptosis in rats with diabetic foot ulcers. Thus, naringin can protect injured endothelial cells and enhance angiogenesis. Presently, naringin enhanced tube formation in HUVECs. As shown in our previous studies, naringin enhanced angiogenesis in vivo and *in vitro*.^31^, ^32^

Bone active and continuous remodelling occurs in response to physiological loading. An ideal scaffold material for bone tissue engineering requires balanced rates of scaffold degradation and tissue regeneration. During the initial stages of scaffold implantation, the scaffold should possess sufficient strength and stiffness to support in vivo tissue ingrowth. In the later stages of bone tissue repair, scaffold degradation provides extra space for improving bone tissue regeneration. A major limitation of this study was that we did not perform an expanded follow‐up study to identify the degree of matching between scaffold degradation and bone tissue growth.

## 5 CONCLUSIONS

A novel porous composite scaffold was fabricated using naringin, SF and HAp. The SF/HAp scaffold incorporated with naringin exhibited favourable biodegradability, biocompatibility, and osteoinductivity in vitro and in vivo. These results indicate the potential usefulness of NG/SF/HAp for bone defect repair and as a degradable implant for clinical orthopaedics.

## CONFLICT OF INTEREST

None.

## AUTHOR CONTRIBUTIONS

Zhihu Zhao and Xinlong Ma designed this study and analysed the data. Bin Zhao, Peng Tian, Jian‐xiong Ma, Jia‐yu Kang, Yang Zhang and Yue Guo involved in responsible of the experiment and figures. Lei Sun performed the μCT. All authors contributed to write and review the manuscript.

## Supporting information

Figure S1

Figure S2

Figure S3

Figure S4

Figure S5

## Data Availability

The data sets generated and analysed during the current study are available from the corresponding author on reasonable request.
