# Supplementary figures and images for: Naringin‐inlaid silk fibroin/hydroxyapatite scaffold enhances human umbilical cord‐derived mesenchymal stem cell‐based bone regeneration
Source: Cell Prolif. 2021 May 19;54(7):e13043. doi: 10.1111/cpr.13043 (PMC8249788; doi:10.1111/cpr.13043)

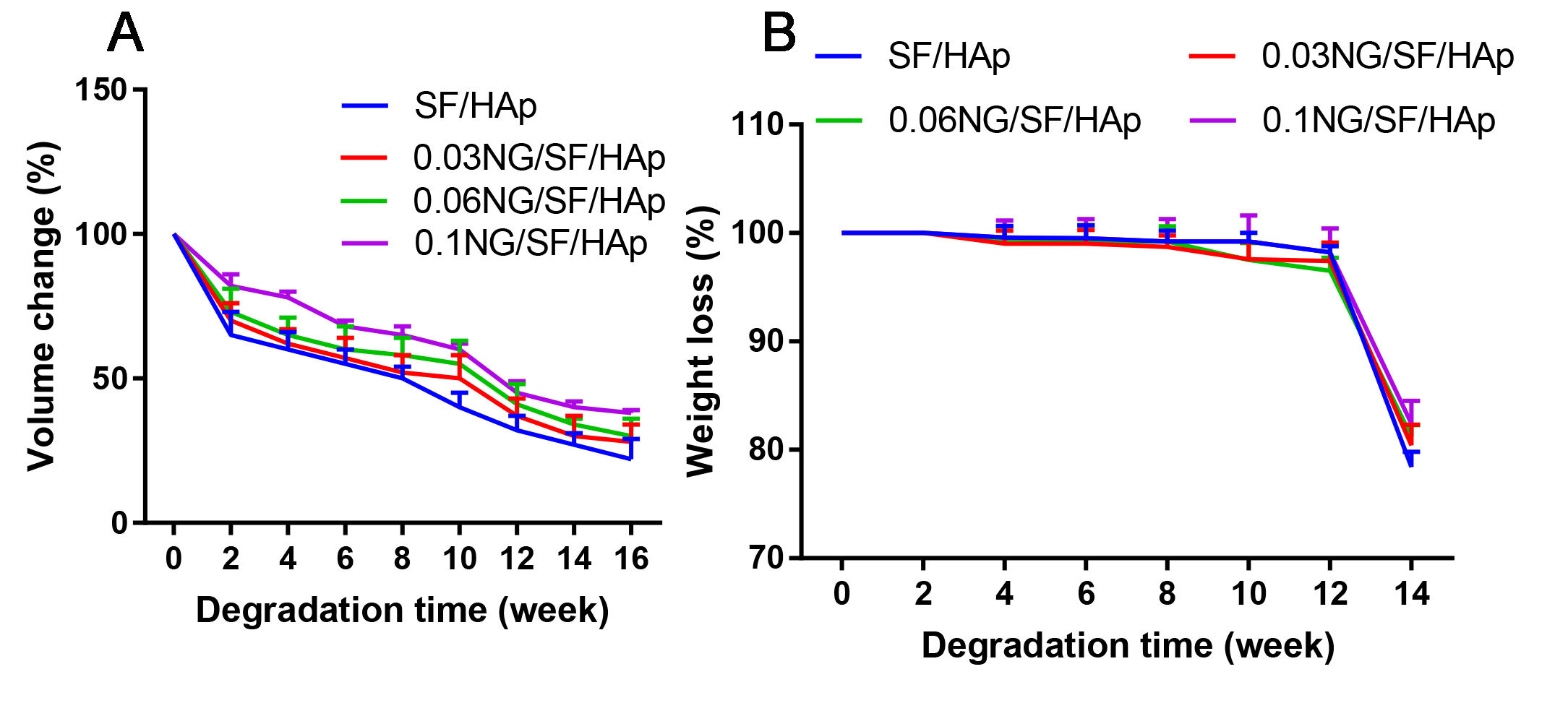

Supplement: Supplementary file 2 — Figure S2 [file CPR-54-e13043-s002.tif]

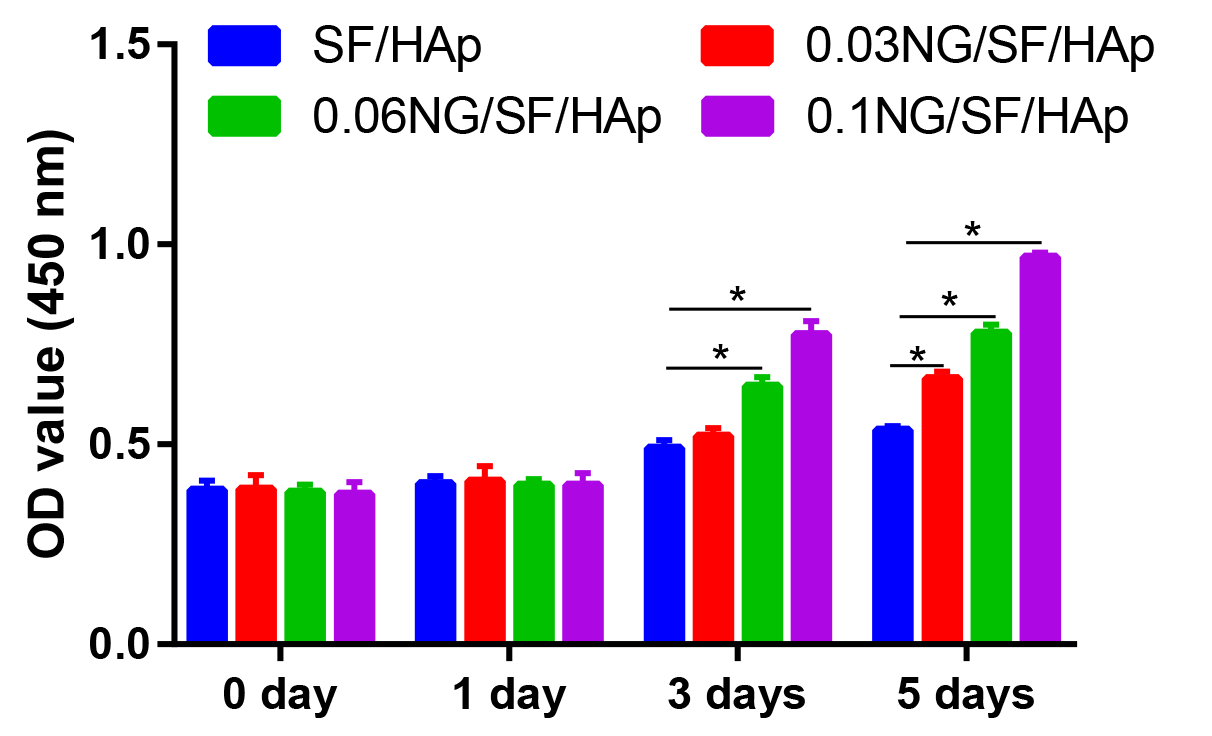

Supplement: Supplementary file 3 — Figure S3 [file CPR-54-e13043-s005.tif]

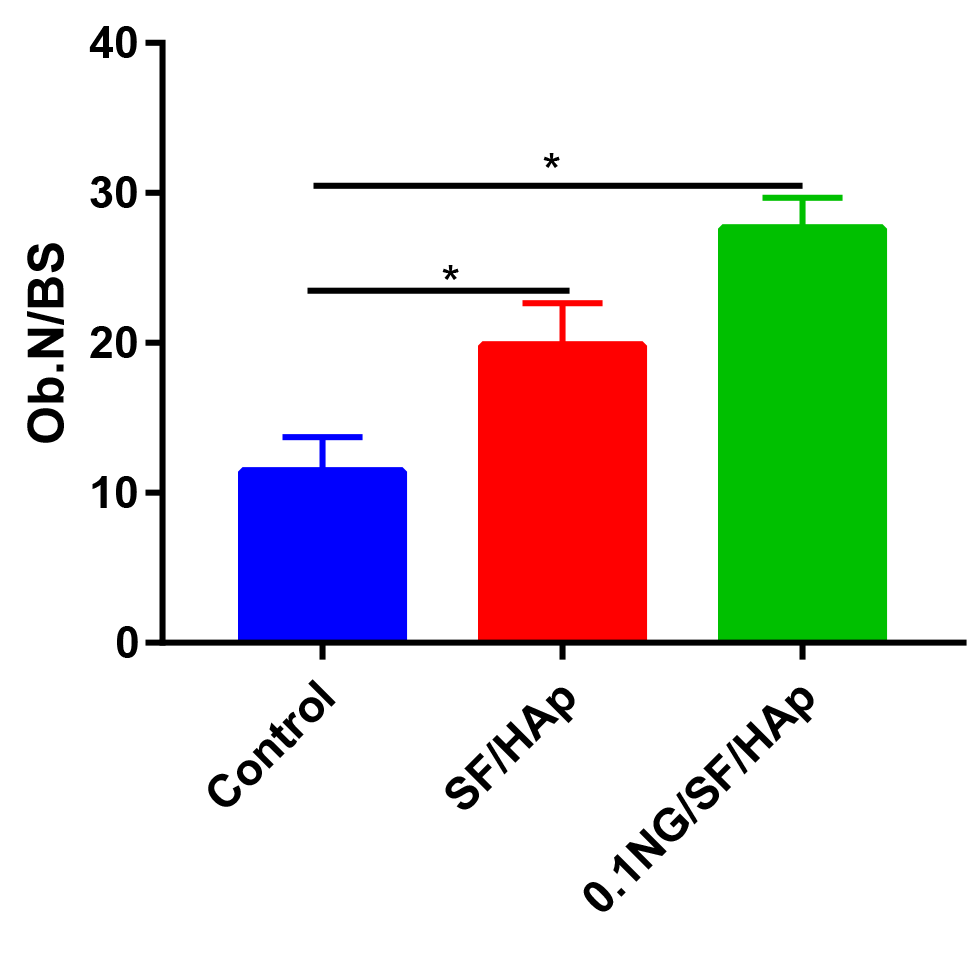

Supplement: Supplementary file 4 — Figure S4 [file CPR-54-e13043-s003.tif]

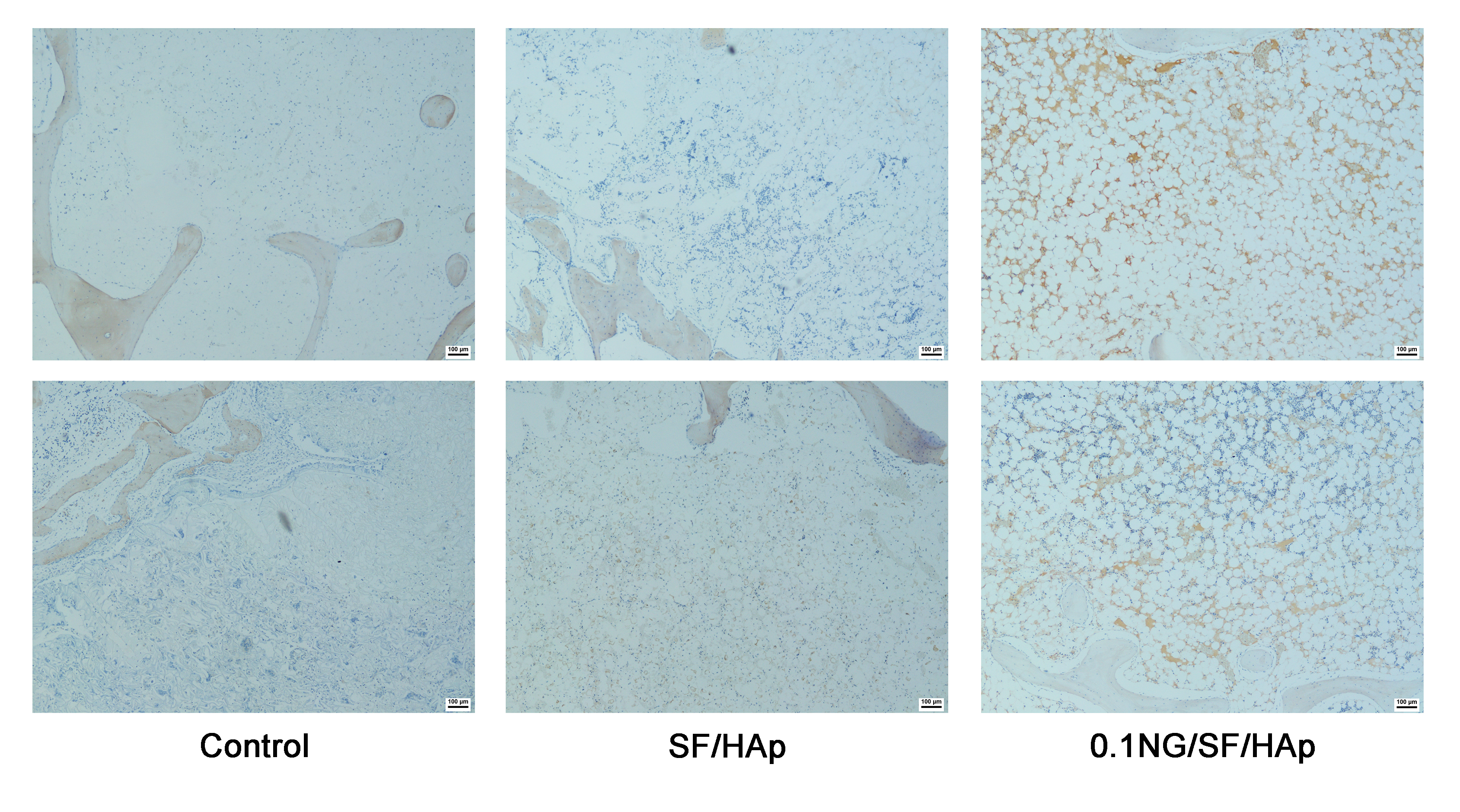

Supplement: Supplementary file 5 — Figure S5 [file CPR-54-e13043-s004.tif]
